# Distributed leadership and teachers’ work engagement: subjective well-being as a mediator and perceived collective teacher efficacy as a moderator

**DOI:** 10.3389/fpsyg.2026.1887759

**Published:** 2026-09-23

**Authors:** Müslim Alanoğlu, Rebin Salih Khudhur, Zülfü Demirtaş, Alper Uslukaya

**Affiliations:** 1 Department of Educational Sciences, Faculty of Education, Firat University, Elazig, Türkiye; 2 Van Yuzuncu Yil Universitesi, Van, Türkiye

**Keywords:** distributed leadership, educational leadership, perceived collective teacher efficacy, well-being, work engagement

## Abstract

**Introduction:**

This study examined the association between distributed leadership and teachers’ work engagement, with teacher subjective well-being specified as a mediator and perceived collective teacher efficacy as a moderator.

**Methods:**

Drawing on the Job Demands–Resources model and social cognitive theory, a moderated mediation model was tested using Bayesian structural equation modeling. Data were collected from 598 teachers working in 60 public schools in Erbil, Kurdistan Region of Iraq.

**Results:**

Distributed leadership was positively associated with teacher subjective well-being [*β* = 0.233, 95% BCI (0.077, 0.381)] and work engagement [*β* = 0.234, 95% BCI (0.135, 0.333)], while subjective well-being was positively associated with work engagement [*β* = 0.172, 95% BCI (0.112, 0.232)]. The indirect association through subjective well-being was supported [*B* = 0.039, 95% BCI (0.012, 0.074)]. Perceived collective teacher efficacy moderated the association between distributed leadership and subjective well-being [*β* = 0.099, 95% BCI (0.003, 0.192)]. The conditional indirect association was supported at the mean and higher levels of perceived collective teacher efficacy but not at lower levels, and the index of moderated mediation excluded zero [*B* = 0.019, 95% BCI (0.001, 0.043)]. These conclusions remained stable under alternative prior specifications.

**Discussion:**

The findings indicate an indirect statistical association between distributed leadership and work engagement through teacher subjective well-being, which varied according to perceived collective teacher efficacy. Given the cross-sectional, self-report design, this mediation represents a statistical decomposition of covariance rather than evidence of a causal mechanism or temporal sequence.

## 1 Introduction

Improving teacher practice and student achievement is a central goal of educational institutions and can be supported through effective organizational processes and leadership practices (Leithwood et al., 2020; Robinson et al., 2008). Although instructional quality is central to school effectiveness, broader organizational and psychological factors, including teachers’ motivation, well-being, and collaborative capacity, also play a critical role in shaping school outcomes (Hoy et al., 2006; Scheerens, 2016). Within this broader perspective, leadership is widely recognized as a key factor associated with school performance and teacher-related outcomes (Grissom et al., 2021; Hallinger, 2011). This issue is particularly relevant in centralized education systems, where teachers’ access to autonomy, participation in decision-making, collegial support, and other organizational resources may depend substantially on how leadership is enacted at the school level (Alanoğlu et al., 2026).

In recent decades, leadership research has shifted from individual-centered perspectives toward more collaborative and shared approaches, highlighting the relevance of distributed leadership in complex school settings (Gronn, 2002; Spillane, 2006). Distributed leadership conceptualizes leadership as a shared and interactive process distributed across multiple actors rather than being confined to a single formal leader (Spillane et al., 2004). This perspective emphasizes interactions among principals, teachers, and other school members in shaping organizational processes and school improvement (Harris, 2013; Leithwood et al., 2009). Empirical studies have consistently shown that distributed leadership is positively associated with teacher outcomes such as work engagement, well-being, and collective efficacy (Hulpia et al., 2011; Liu et al., 2021). By expanding teachers’ participation in decision-making and strengthening collaboration and shared responsibility, distributed leadership may operate as an organizational resource through which teachers experience greater support, professional influence, and belonging. Evidence from a centralized education system, indicates that distributed leadership is positively associated with teachers’ subjective well-being and that this association becomes stronger when teachers possess greater psychological resources (Çevik, 2025).

Increasing attention has also been given to teachers’ psychological functioning in educational research. Work engagement, defined as a positive and fulfilling work-related state characterized by vigor, dedication, and absorption (Schaufeli et al., 2002), has been associated with instructional quality and student outcomes. Similarly, subjective well-being, encompassing emotional, psychological, and professional dimensions, has emerged as a key factor for sustainable school improvement (Collie et al., 2012; Diener et al., 2018). Leadership practices that promote support, shared responsibility, and professional autonomy are associated with more favorable psychological and motivational outcomes among teachers (Bakker and Demerouti, 2017; Nielsen and Daniels, 2012). In this respect, subjective well-being may constitute a psychological mechanism connecting teachers’ perceptions of their organizational and leadership environment with their willingness to invest energy and effort in their work. KRG-based evidence has linked supportive school conditions and psychological empowerment to teacher well-being, but distributed leadership and work engagement have not been incorporated into this explanatory framework (Alanoğlu et al., 2026).

Another construct closely related to school effectiveness is collective teacher efficacy, which refers to teachers’ shared beliefs in their collective ability to positively influence student learning (Bandura, 1997; Goddard et al., 2004). Collective teacher efficacy can therefore be conceptualized not merely as an outcome of leadership but as a contextual psychological resource that may strengthen the positive association between distributed leadership and teachers’ subjective well-being.

Despite evidence indicating that distributed leadership is positively associated with teacher outcomes, the underlying mechanisms and contextual conditions of these relationships remain insufficiently understood (Liu et al., 2021; Tian et al., 2016). In particular, limited attention has been paid to how distributed leadership is associated with work engagement through teachers’ psychological functioning (i.e., well-being) and under what conditions these associations become stronger or weaker. This gap is especially salient in the KRG, where a tension between policy and practice remains: although recent legislation has established formal school-level structures for staff participation, evidence from Erbil indicates that teachers continue to report relatively limited experiences of distributed leadership (Demirtaş and Khudhur, 2026; Kurdistan Parliament, 2022). Existing KRG research has linked school culture and psychological empowerment to teachers’ subjective well-being (Alanoğlu et al., 2026), whereas recent leadership studies have largely focused on transformational leadership and organizational outcomes in higher education (Abdullah Kakahmed and Maroof Jameel, 2026). Consequently, the available evidence does not explain whether distributed leadership is indirectly associated with teachers’ work engagement through subjective well-being or whether this indirect association varies across levels of collective teacher efficacy.

The Job Demands–Resources (JD-R) model provides the rationale for conceptualizing distributed leadership as an organizational resource associated with teachers’ psychological functioning and work engagement. Social cognitive theory complements this explanation by suggesting that shared efficacy beliefs influence how teachers interpret organizational opportunities and mobilize collective resources. Integrating these perspectives makes it possible to explain both the psychological mechanism through which distributed leadership is related to work engagement and the collective condition under which this mechanism may be stronger.

Accordingly, this study examines the relationships between distributed leadership and teachers’ work engagement by proposing a moderated mediation model in which subjective well-being serves as a mediator and collective teacher efficacy functions as a moderator. The study makes three contributions. First, it examines subjective well-being as a psychological mechanism linking distributed leadership to work engagement rather than treating well-being solely as an outcome. Second, it conceptualizes collective teacher efficacy as a boundary condition that may alter the strength of the association between distributed leadership and subjective well-being. Third, it tests these relationships in the KRG, a centralized and hierarchically structured educational setting in which school-level leadership practices may be particularly consequential for teachers’ access to participation, support, and professional influence. In this way, the study moves beyond asking whether distributed leadership is associated with favorable teacher outcomes and examines how, under what conditions, and within which institutional context these associations emerge.

## 2 The context of the Kurdistan region of Iraq

The Kurdistan Region of Iraq (KRG) education system operates within a semi-autonomous governance structure in which educational policy and administration are largely regulated by the regional Ministry of Education while remaining aligned with the broader Iraqi education system. Despite some school-level autonomy in instructional supervision and internal management, key decisions concerning curriculum design, staffing, leadership appointments, and assessment remain largely under ministerial control (Alanoğlu et al., 2026; Vernez et al., 2014).

School leadership in the KRG has traditionally been characterized by hierarchical and bureaucratic structures, with principals expected to fulfil both administrative and instructional leadership roles (Vernez et al., 2014). School leaders therefore occupy a critical position between centrally determined requirements and teachers’ everyday professional experiences. Although centralized governance can promote consistency across schools, limited autonomy and hierarchical decision-making may restrict teacher participation, professional influence, and collaborative responsibility (Alanoğlu et al., 2026). Distributed leadership is particularly relevant in this context because it may expand teacher participation, collegial support, and shared responsibility without displacing formal authority.

Recent legislative developments nevertheless indicate an effort to create greater scope for school-level participation. The KRG Education Law No. 13 of 2022 introduced formal provisions concerning staff councils and school committees, creating institutional structures through which teachers may participate in school governance (Kurdistan Parliament, 2022). However, evidence from 488 teachers in Erbil public schools showed relatively low perceptions of distributed leadership across bounded empowerment, leadership development, shared decision-making, and collective engagement (Demirtaş and Khudhur, 2026). This contrast suggests that formal participatory structures may not yet have translated into substantive experiences of shared influence and responsibility among teachers.

Leadership scholarship in the KRG has also examined transformational leadership in relation to organizational outcomes in higher education, including strategic capacity (Abdullah Kakahmed and Maroof Jameel, 2026). However, this study does not address distributed leadership or teachers’ psychological outcomes in public schools.

At the school level, supportive school cultures in Erbil have been positively associated with teachers’ subjective well-being through psychological empowerment (Alanoğlu et al., 2026). Nevertheless, school culture and distributed leadership are conceptually distinct, and existing evidence does not explain how the distribution of leadership activity is associated with teachers’ well-being and work engagement. Taken together, the coexistence of centralized governance, formally established participatory structures, and teachers’ relatively limited experiences of distributed leadership makes the KRG a particularly relevant context for examining these relationships.

## 3 Literature review and hypotheses

This study draws on distributed leadership, the JD-R model, and social cognitive theory. Distributed leadership explains how leadership activity and influence extend beyond a single formal leader and emerge through interactions among school members (Gronn, 2002; Spillane et al., 2004). From a JD-R perspective, participatory decision-making, shared responsibility, and mutual support may function as organizational resources associated with teachers’ positive functioning and willingness to invest energy in their work (Bakker and Demerouti, 2017). Social cognitive theory adds that beliefs about collective capability shape how individuals interpret and respond to organizational conditions (Bandura, 1997). Together, these perspectives provide the basis for the direct, indirect, and conditional associations proposed in this study.

### 3.1 Distributed leadership as a participatory organizational resource

Distributed leadership challenges leader-centered accounts by locating leadership in the interactions among leaders, followers, and their situation (Spillane et al., 2004). It does not simply mean delegating administrative tasks; it concerns the distribution of influence, participation, and responsibility across organizational members. The concept is broader, however, than any single set of indicators. In the present study, emphasis is placed primarily on participatory decision-making, shared responsibility, and a collaborative culture of mutual support (Bellibaş et al., 2024; Spillane et al., 2004). This conceptual focus captures central elements of distributed leadership without encompassing all relational and practice-based dimensions included in broader theoretical accounts of the construct.

Participation, professional voice, shared responsibility, and mutual support may reduce teachers’ sense of isolation and increase their access to social and organizational resources. Research based on TALIS data has associated distributed leadership with supportive school culture, teacher collaboration, self-efficacy, and job satisfaction (Liu et al., 2021). International evidence has also linked distributed leadership to teacher commitment through workload stress and teacher well-being, although the strength of these pathways varies across education systems (Bellibaş et al., 2024).

### 3.2 Distributed leadership and teacher subjective well-being

In the present study, teacher subjective well-being is conceptualized as teachers’ positive psychological functioning within their work context rather than as a broad evaluation of general happiness, life satisfaction, or the absence of psychological distress. This work-specific conceptualization comprises school connectedness and teaching efficacy (Renshaw et al., 2015). School connectedness refers to feeling supported by, belonging to, and relating well with others at school, whereas teaching efficacy reflects teachers’ appraisal of their capacity to meet professional demands effectively. Together, these dimensions represent teachers’ perceived relational and competence-related functioning at work.

From a JD-R perspective, distributed leadership may be associated with this form of well-being because meaningful participation and mutual support provide relational and organizational resources. Opportunities to contribute to decisions may affirm teachers’ professional voice, while shared responsibility and collaborative support may strengthen belonging and confidence in addressing teaching demands. Consistent with this reasoning, Liu et al. (2024) found that distributed leadership was indirectly associated with teacher well-being through organizational trust, indicating that the relationship may depend on the relational conditions through which leadership is experienced. Bellibaş et al. (2024), using TALIS data from 47 education systems, found that teacher well-being mediated part of the association between distributed leadership and teacher commitment. Çevik (2025) reported a positive association between distributed leadership and teacher subjective well-being. These findings do not imply that distributed leadership invariably produces well-being, but they support the expectation that stronger perceptions of participatory and collaborative leadership are associated with more favorable work-specific psychological functioning.

*H1: Distributed leadership is positively associated with teacher subjective well-being.*

### 3.3 Distributed leadership and work engagement

Work engagement is a positive and fulfilling work-related state comprising vigor, dedication, and absorption (Schaufeli et al., 2006). Vigor reflects energy, resilience, and persistence at work; dedication denotes significance, enthusiasm, inspiration, and pride; and absorption refers to being deeply concentrated and engrossed in one’s work. Although vigor, dedication, and absorption are conceptually distinguishable, they are strongly interrelated and jointly represent the broader construct of work engagement (Schaufeli et al., 2006).

The JD-R model proposes that job resources promote engagement because they help employees accomplish work goals, reduce the psychological costs of demands, and stimulate motivation (Bakker and Demerouti, 2017). Distributed leadership may provide such resources by increasing professional voice, access to collegial support, and meaningful involvement in school affairs. When teachers perceive that participation is genuine and responsibility is shared, they may experience greater ownership of school goals and stronger reasons to invest effort in their work. Previous distributed-leadership studies have linked participatory and collaborative leadership practices to related work attitudes, including organizational commitment and job satisfaction (Hulpia et al., 2009; Liu et al., 2021; Bellibaş et al., 2024). The proposed association with work engagement extends this evidence by focusing specifically on teachers’ energy, involvement, and persistence at work.

*H2: Distributed leadership is positively associated with teachers’ work engagement.*

### 3.4 Subjective well-being, work engagement, and the mediating mechanism

Teacher subjective well-being and work engagement are related but not interchangeable. In this study, subjective well-being reflects teachers’ perceived connectedness to their school and confidence in their own teaching, whereas work engagement reflects the energy, dedication, and absorption invested in work. This distinction provides the basis for the proposed ordering. Teachers who feel connected to their school and confident in meeting teaching demands may have the relational support and professional confidence needed to sustain effort in their work. School connectedness can make work socially meaningful, while teaching efficacy can increase willingness to persist when work is demanding. Work engagement is therefore positioned as a motivational state that may follow from positive work-specific functioning rather than as another indicator of the same construct.

Empirical evidence supports an association between the two constructs, although their temporal ordering remains open to question. Kalman (2026) found that teacher subjective well-being, comprising school connectedness and teaching efficacy, was positively associated with work engagement. A meta-analysis likewise identified work engagement as one of the strongest correlates of teacher well-being (Zhou et al., 2024). Given the cross-sectional design of the present study, reciprocal relationships cannot be excluded; engagement may also contribute to teachers’ efficacy and connectedness over time. H3 therefore specifies the theoretically proposed direction of the association without claiming temporal or causal precedence.

*H3: Teacher subjective well-being is positively associated with work engagement.*

This distinction also clarifies the mediation mechanism. Distributed leadership represents an organizational condition, whereas subjective well-being reflects teachers’ positive psychological functioning within that condition. Participatory decision-making and mutual support may be associated with stronger school connectedness and teaching efficacy, which may, in turn, be associated with greater vigor, dedication, and absorption. Within the JD-R model, subjective well-being is therefore proposed to carry part of the association between an organizational resource and work engagement. Related evidence has placed teacher well-being in indirect pathways linking distributed leadership to commitment (Bellibaş et al., 2024) and professional satisfaction (Çevik, 2025). The present study examines whether a similar indirect association extends to work engagement.

*H4: Distributed leadership is indirectly associated with work engagement through teacher subjective well-being.*

### 3.5 The moderating role of perceived collective teacher efficacy

Collective teacher efficacy refers to beliefs that teachers in a school, as a group, can organize and execute the actions required to influence student learning (Bandura, 1997; Goddard et al., 2004). Although collective teacher efficacy is inherently group-referent, the present study examines individual teachers’ perceptions of the collective capability of teachers in their school rather than an aggregated school-level construct. It is therefore interpreted as perceived collective teacher efficacy.

Teaching efficacy within the subjective well-being construct and perceived collective teacher efficacy are conceptually distinct. Teaching efficacy concerns teachers’ appraisal of their own teaching capability, whereas perceived collective teacher efficacy concerns their appraisal of the shared capability of teachers in their school. The distinction between an individual self-belief and a group-referent belief is central to social cognitive theory, even though the two may be empirically related (Bandura, 1997; Goddard et al., 2004). This distinction is particularly important here because perceived collective teacher efficacy is proposed to condition the association between distributed leadership and individual well-being.

Leadership practices may contribute to collective efficacy over time by creating opportunities for collaboration and shared success. However, social cognitive theory also recognizes reciprocal relationships among beliefs, behavior, and environmental conditions (Bandura, 1997). Accordingly, the present model treats teachers’ existing perceptions of collective capability as a condition that may shape how strongly perceived distributed leadership is associated with their well-being.

An amplifying rather than compensatory pattern is expected because distributed leadership requires collective capacity to convert participation into coordinated practice. When teachers believe that teachers in their school can jointly address instructional and behavioral challenges, shared influence and responsibility are more likely to be accompanied by coordinated action, mutual support, and mastery experiences. These conditions may strengthen the association of distributed leadership with connectedness and teaching efficacy. Conversely, when perceived collective efficacy is weak, distributed responsibility may be experienced as symbolic participation, uncoordinated responsibility, or an additional demand. Although collective efficacy could theoretically compensate for weak leadership, amplification is expected because the benefits of distributed leadership depend on confidence that teachers can enact shared responsibilities effectively.

*H5: Perceived collective teacher efficacy moderates the positive association between distributed leadership and teacher subjective well-being, such that the association is stronger among teachers reporting higher perceived collective teacher efficacy.*

### 3.6 The conditional indirect association

Together, the JD-R model and social cognitive theory suggest a conditional indirect association. If perceived collective teacher efficacy strengthens the association between distributed leadership and teacher subjective well-being, the subsequent indirect association with work engagement should also become stronger. Accordingly, the extent to which participatory leadership is indirectly associated with work engagement may depend partly on teachers’ confidence that teachers in their school can enact shared influence and responsibility effectively.

*H6: The positive indirect association between distributed leadership and work engagement through teacher subjective well-being is stronger among teachers reporting higher perceived collective teacher efficacy.*

Figure 1 presents the hypothesized moderated mediation model tested in this study.

**Figure 1:**
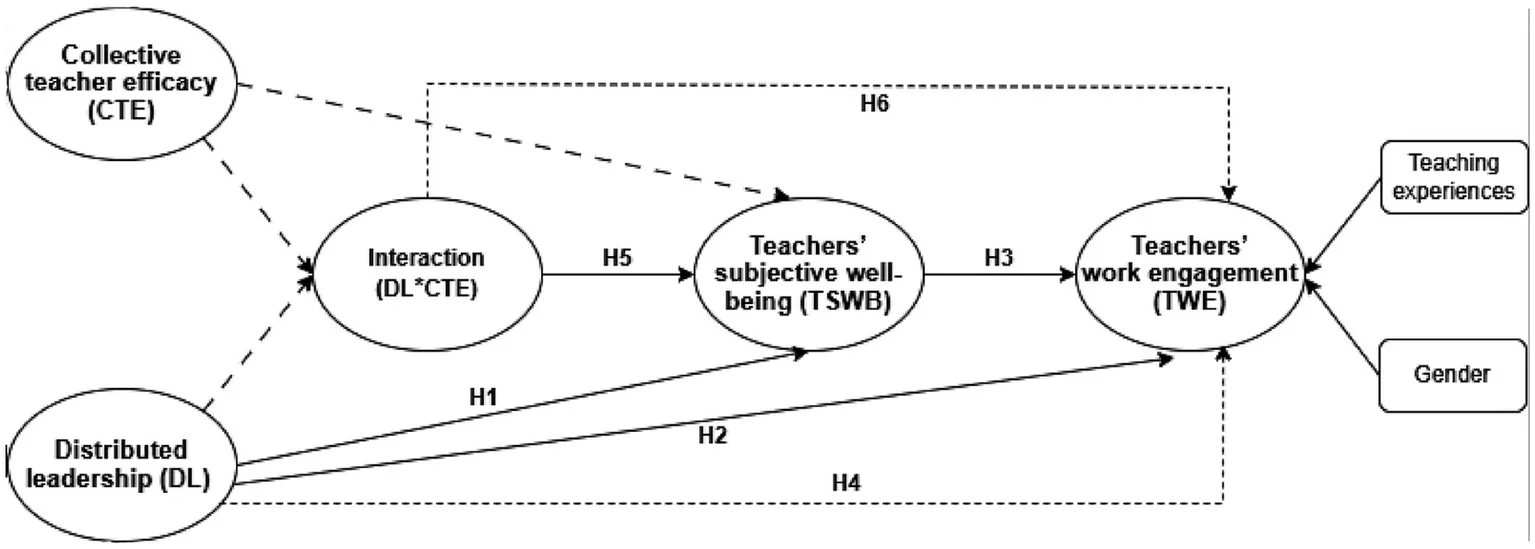
Conceptual model and hypothesis.

## 4 Method

### 4.1 Research design

This study employed a quantitative, cross-sectional survey design. The hypothesized relationships among distributed leadership, teacher subjective well-being, work engagement, and perceived collective teacher efficacy were examined using structural equation modeling (SEM). SEM is a multivariate statistical technique that enables the examination of complex relationships among observed variables and latent constructs within a theoretically specified model (Byrne, 2013; Hayduk et al., 2005). More specifically, a latent moderated mediation model was estimated to test the indirect association between distributed leadership and work engagement through teacher subjective well-being and whether this indirect association varied according to perceived collective teacher efficacy. Given the cross-sectional design, the specified directions represent theoretically proposed associations and do not establish temporal ordering or causality.

### 4.2 Participants and procedure

The analytical sample comprised 598 teachers working in 60 public schools in Erbil, Kurdistan Region of Iraq, during the 2025–2026 academic year. Convenience sampling was used because access to participating schools depended on institutional availability. The online questionnaire was circulated to all teachers through the participating schools’ existing communication networks rather than being directed to teachers individually selected by school principals. Because the total number of teachers who received the survey link was not systematically recorded, an overall response rate and school-specific response rates could not be calculated.

Ethical approval was obtained from the Fırat University Social and Human Sciences Research Ethics Committee before data collection (meeting no. 2026/5, decision no. 790302). Data were subsequently collected through Google Forms between March and April 2026. A total of 606 questionnaires were returned. During data screening, eight questionnaires in which at least half of the survey items had not been answered were excluded, resulting in a final analytical sample of 598 teachers. Participation was voluntary and anonymous, and participants provided electronic informed consent before completing the questionnaire.

Of the final sample, 254 teachers (42.5%) were female and 344 (57.5%) were male. Participants worked at the primary (*n* = 250, 41.8%), middle-school (*n* = 179, 29.9%), and high-school (*n* = 169, 28.3%) levels. Their teaching experience ranged from 2 to 16 years (M = 8.94, SD = 3.31).

### 4.3 Measures

This study included four main constructs: distributed leadership, teacher subjective well-being, work engagement, and collective teacher efficacy. Although the instruments were administered in their original English-language versions and their factorial structures were supported in the present sample, English may not have been the first language of all participants. Possible differences in language proficiency and item interpretation should therefore be considered when interpreting the findings.

#### 4.3.1 Collective teacher efficacy

Perceived collective teacher efficacy was specified as the moderating variable and was assessed through individual teachers’ perceptions of their colleagues’ collective capability to influence student learning. The Collective Teacher Efficacy Scale, originally developed by Tschannen-Moran and Barr (2004), was used in this study. The instrument consists of 12 items grouped into two subdimensions: Student Discipline (e.g., “How clearly can teachers at your school communicate the expected behaviors to students?”) and Instructional Strategies (e.g., “To what extent can teachers at your school foster students’ critical thinking?”). Responses were rated on a five-point Likert scale ranging from 1 (not at all) to 5 (completely). CFA results indicated that the model demonstrated good fit to the data, *χ*^2^(52) = 151.314, *p* < 0.001, RMSEA = 0.057, CFI = 0.977, TLI = 0.970, and SRMR = 0.024.

#### 4.3.2 Distributed leadership

Distributed leadership was specified as the main independent variable. It was assessed using five items from the Teaching and Learning International Survey (TALIS) teacher questionnaire developed by the Organisation for Economic Co-operation and Development (OECD, 2018; T3STAKE). These items capture teachers’ perceptions of stakeholder involvement in decision-making, shared responsibility, and collaborative school culture (e.g., “This school provides staff with opportunities to actively participate in school decisions”). Accordingly, the measure operationalizes the participatory decision-making, shared-responsibility, and collaborative-culture components of distributed leadership rather than every relational and practice-based dimension included in broader conceptualizations of the construct. Responses were recorded on a four-point Likert scale ranging from 1 (strongly disagree) to 4 (strongly agree). CFA results indicated that the model demonstrated good fit to the data, *χ*^2^(4) = 16.026, *p* = 0.003, RMSEA = 0.071, CFI = 0.992, TLI = 0.979, and SRMR = 0.016.

#### 4.3.3 Teacher subjective well-being

Teacher subjective well-being was specified as the mediating variable. It was assessed using the Teacher Subjective Well-Being Questionnaire (TSWQ) developed by Renshaw et al. (2015), which consists of eight items reflecting two dimensions: teaching efficacy (e.g., “I am a successful teacher”) and school connectedness (e.g., “I am treated with respect at this school”). Thus, teacher subjective well-being was operationalized as teachers’ positive work-specific functioning reflected in teaching efficacy and school connectedness, rather than as general happiness, life satisfaction, or the absence of psychological distress. Participants rated each item based on their experiences within their school context. Responses were provided on a four-point Likert scale ranging from 1 (almost never) to 4 (almost always). CFA results indicated that the model demonstrated good fit to the data, *χ*^2^(18) = 78.512, *p* < 0.001, RMSEA = 0.075, CFI = 0.975, TLI = 0.961, and SRMR = 0.027.

#### 4.3.4 Work engagement

Teacher work engagement was specified as the dependent (outcome) variable. The nine-item short version of the Utrecht Work Engagement Scale (UWES-9) was used in this study. Originally developed by Schaufeli et al. (2002) and later psychometrically validated by Schaufeli et al. (2006), the scale comprises three dimensions: vigor (e.g., “When I get up in the morning, I feel like going to work”), dedication (e.g., “I am proud of the work that I do”), and absorption (e.g., “I get carried away when I’m working”). Responses were provided on a seven-point Likert scale ranging from 1 (almost never) to 7 (always).

CFA results indicated that the model demonstrated excellent fit to the data, *χ*^2^(24) = 49.176, *p* = 0.002, RMSEA = 0.042, CFI = 0.993, TLI = 0.990, and SRMR = 0.013.

#### 4.3.5 Control variables

Control variables included teaching experience and gender. Teaching experience was treated as a continuous variable ranging from 2 to 16 years (M = 8.94). Gender was dummy coded (0 = female, 1 = male). Both variables were included as covariates predicting teacher work engagement in the structural model.

### 4.4 Data analysis

Before the main analyses, the dataset was screened, and preliminary analyses were performed. Univariate normality was assessed by examining skewness and kurtosis values, and values within the range of ±2 were considered indicative of acceptable normality (George and Mallery, 2016). To assess multicollinearity among the predictor variables, variance inflation factor (VIF) and tolerance values were calculated. VIF values below 5 and tolerance values above 0.20 indicated that multicollinearity was not a concern (Hair et al., 2019).

Given that all data were collected through self-report measures at a single time point, the potential risk of common method bias was evaluated. Harman’s single-factor test indicated that the first unrotated factor accounted for 45.301% of the total variance. Because Harman’s test provides only a limited diagnostic of common method bias, a CFA-based single-factor assessment was also conducted. The model in which all 34 items were constrained to load on a single latent factor demonstrated poor fit to the data, robust *χ*^2^(527) = 5060.096, *p* < 0.001, RMSEA = 0.120, 90% CI [0.117, 0.123], CFI = 0.680, TLI = 0.660, and SRMR = 0.117. These findings indicated that a single common factor did not adequately account for the covariance among the study measures. In addition, procedural remedies, including ensuring participant anonymity and using different response formats across the measures, were implemented during data collection to reduce potential bias (Podsakoff et al., 2003). Nevertheless, because all focal variables were obtained from teacher self-reports at a single time point, common method bias could not be completely ruled out.

The reliability and validity of the measurement instruments were evaluated using confirmatory factor analysis (CFA). Internal consistency reliability was assessed using Cronbach’s alpha coefficients, with values above 0.70 considered acceptable (Nunnally and Bernstein, 1994). Convergent validity was examined using composite reliability (CR) and average variance extracted (AVE), with CR values exceeding 0.70 and AVE values exceeding 0.50 indicating adequate convergent validity (Fornell and Larcker, 1981). Discriminant validity was initially assessed by comparing the square root of each construct’s AVE with its correlations with the other constructs. Given the marginal Fornell–Larcker result for distributed leadership and collective teacher efficacy, discriminant validity was additionally examined using the heterotrait–monotrait ratio of correlations (HTMT), with values below 0.85 considered evidence of adequate discriminant validity (Henseler et al., 2015).

Before testing the structural model, the measurement model was evaluated using the robust maximum likelihood (MLR) estimator. Distributed leadership and teacher subjective well-being were specified as first-order latent constructs. Collective teacher efficacy was modeled as a second-order latent construct comprising student discipline and instructional strategies, while work engagement was modeled as a second-order latent construct comprising vigor, dedication, and absorption. Model fit was evaluated using the Comparative Fit Index (CFI), Tucker–Lewis Index (TLI), Root Mean Square Error of Approximation (RMSEA), and Standardized Root Mean Square Residual (SRMR). CFI and TLI values above 0.90 and RMSEA and SRMR values below 0.08 were considered indicative of acceptable model fit (Browne and Cudeck, 1992).

The structural model was estimated using a Bayesian approach. Although MLR was used to evaluate the measurement model and provide conventional global fit indices, Bayesian estimation was employed for the latent moderated mediation model because it permits the direct estimation of posterior distributions and credible intervals for latent interaction and conditional indirect effects (Muthén and Asparouhov, 2012; Van de Schoot et al., 2014). Prior selection was guided by the availability of parameter-specific evidence. Because no prior KRG research or closely comparable study provided transferable estimates for the latent interaction and moderated mediation parameters, informative priors could not be justified. These parameters were exploratory in terms of their expected magnitudes; therefore, Mplus diffuse priors were used as a data-dominant baseline (Muthén and Asparouhov, 2012; Zyphur and Oswald, 2015). Because default diffuse priors are not necessarily neutral, prior sensitivity was examined using weakly informative *N*(0,1) and broader *N*(0,10) priors (Depaoli et al., 2020; Smid and Winter, 2020). The direction, magnitude, and 95% credible intervals of the focal effects were compared across the three specifications. During model estimation, two parallel Markov chain Monte Carlo (MCMC) chains were run simultaneously, and convergence was assessed using the potential scale reduction (PSR) criterion. PSR values below 1.05 and their stability throughout the estimation process were considered evidence of convergence (Gelman and Rubin, 1992). Convergence and model stability were further evaluated through inspection of trace plots and posterior distributions. Adequate mixing of the chains without systematic trends in the trace plots, together with approximately unimodal and symmetric posterior distributions, was considered additional evidence of convergence (Van de Schoot and Depaoli, 2014).

Direct, indirect, and interaction effects were evaluated using posterior point estimates and 95% Bayesian credible intervals. An effect was considered supported when its 95% credible interval excluded zero. Posterior one-tailed probability values produced by Mplus were reported as supplementary information and were not treated as conventional frequentist *p*-values. Simple slope analyses were conducted at low (−1 SD), mean, and high (+1 SD) levels of collective teacher efficacy (Aiken and West, 1991). Conditional indirect effects were examined at −2 SD, −1 SD, the mean, +1 SD, and +2 SD levels of the moderator. The index of moderated mediation was calculated to determine whether the indirect association between distributed leadership and work engagement through teacher subjective well-being varied systematically as a function of collective teacher efficacy (Hayes, 2015).

## 5 Results

### 5.1 Preliminary analyses

Table 1 presents the means, standard deviations, correlations, and multicollinearity indicators for the variables included in the study.

**Table 1:** Descriptive statistics, correlations, and multicollinearity diagnostics (*N* = 598).

Variable/statistic	M	SD	Skewness	Kurtosis	1	2	3	4
1. DL	3.056	0.771	−1.122	−0.129	**0.794**	0.751**	0.658**	0.234**
2. TCE	3.663	0.965	−1.007	−0.459		**0.732**	0.738**	0.270**
3. WE	4.609	1.148	−1.254	0.287			**0.840**	0.333**
4. WB	3.181	0.728	−1.573	1.021				**0.753**
VIF					2.425	3.017	2.319	—
Tolerance					0.412	0.331	0.431	—
Cronbach’s *α*					0.892	0.949	0.942	0.912
CR					0.895	0.932	0.914	0.913
AVE					0.630	0.535	0.705	0.567

^**^p < 0.01. Bold diagonal values represent the square root of the average variance extracted (AVE).

The descriptive statistics showed that the mean scores were 3.056 (SD = 0.771) for DL, 3.663 (SD = 0.965) for TCE, 4.609 (SD = 1.148) for WE, and 3.181 (SD = 0.728) for WB. Because the constructs were measured using different response scales, their raw mean scores were not directly compared. Skewness values ranged from −1.573 to −1.007, while kurtosis values ranged from −0.459 to 1.021. As all values fell within the recommended range of −2 to +2, the distributions were considered to demonstrate an acceptable approximation to univariate normality. Correlation analysis revealed that all variables were positively and significantly related to each other. Specifically, DL was positively correlated with TCE (*r* = 0.751, *p* < 0.01), WE (*r* = 0.658, *p* < 0.01), and WB (*r* = 0.234, *p* < 0.01), while TCE was also positively associated with WE (*r* = 0.738, *p* < 0.01) and WB (*r* = 0.270, *p* < 0.01). Additionally, WE was positively correlated with WB (*r* = 0.333, *p* < 0.01).

Multicollinearity diagnostics showed that VIF values ranged from 2.319 to 3.017 and tolerance values ranged from 0.331 to 0.431, indicating that multicollinearity was not a serious concern. Furthermore, the reliability analysis demonstrated that Cronbach’s alpha values ranged from 0.892 to 0.949. In addition, CR values ranged from 0.895 to 0.932, and AVE values ranged from 0.535 to 0.705, supporting the internal consistency reliability and convergent validity of the constructs. Discriminant validity was initially assessed using the Fornell–Larcker criterion. While most constructs met this criterion, the square root of AVE for TCE (0.732) was slightly lower than its correlation with DL (*r* = 0.751). Therefore, discriminant validity was additionally evaluated using the HTMT. The HTMT values ranged from 0.294 to 0.788, with the DL–TCE value estimated at 0.788. As all HTMT values were below the conservative threshold of 0.85, the results provided additional support for the empirical distinctiveness of the constructs despite the marginal Fornell–Larcker result for DL and TCE.

### 5.2 Model testing

Before testing the structural model, the hypothesized multidimensional measurement model was evaluated. The measurement model, specified without structural paths among the four focal latent constructs, demonstrated good fit to the data: robust *χ*^2^(516) = 1137.746, *p* < 0.001, RMSEA = 0.045, 90% CI [0.041, 0.048], CFI = 0.956, TLI = 0.952, and SRMR = 0.036.

The hypothesized structural paths were subsequently added to the measurement model and estimated using Bayesian estimation with the default diffuse priors implemented in Mplus. Two parallel Markov chain Monte Carlo (MCMC) chains were run for 70,000 iterations. Convergence was evaluated using the PSR criterion, trace plots, and posterior distribution plots. The maximum PSR value first fell below 1.05 at 56,100 iterations (PSR = 1.049), remained below this threshold thereafter, and reached 1.014 at the final iteration. Visual inspection of the trace plots indicated adequate mixing and no systematic divergence between the chains, while the posterior distributions were generally unimodal and approximately symmetric (Figure 2). Collectively, these diagnostics indicated satisfactory convergence and supported the interpretation of the posterior estimates (Van de Schoot and Depaoli, 2014).

**Figure 2:**
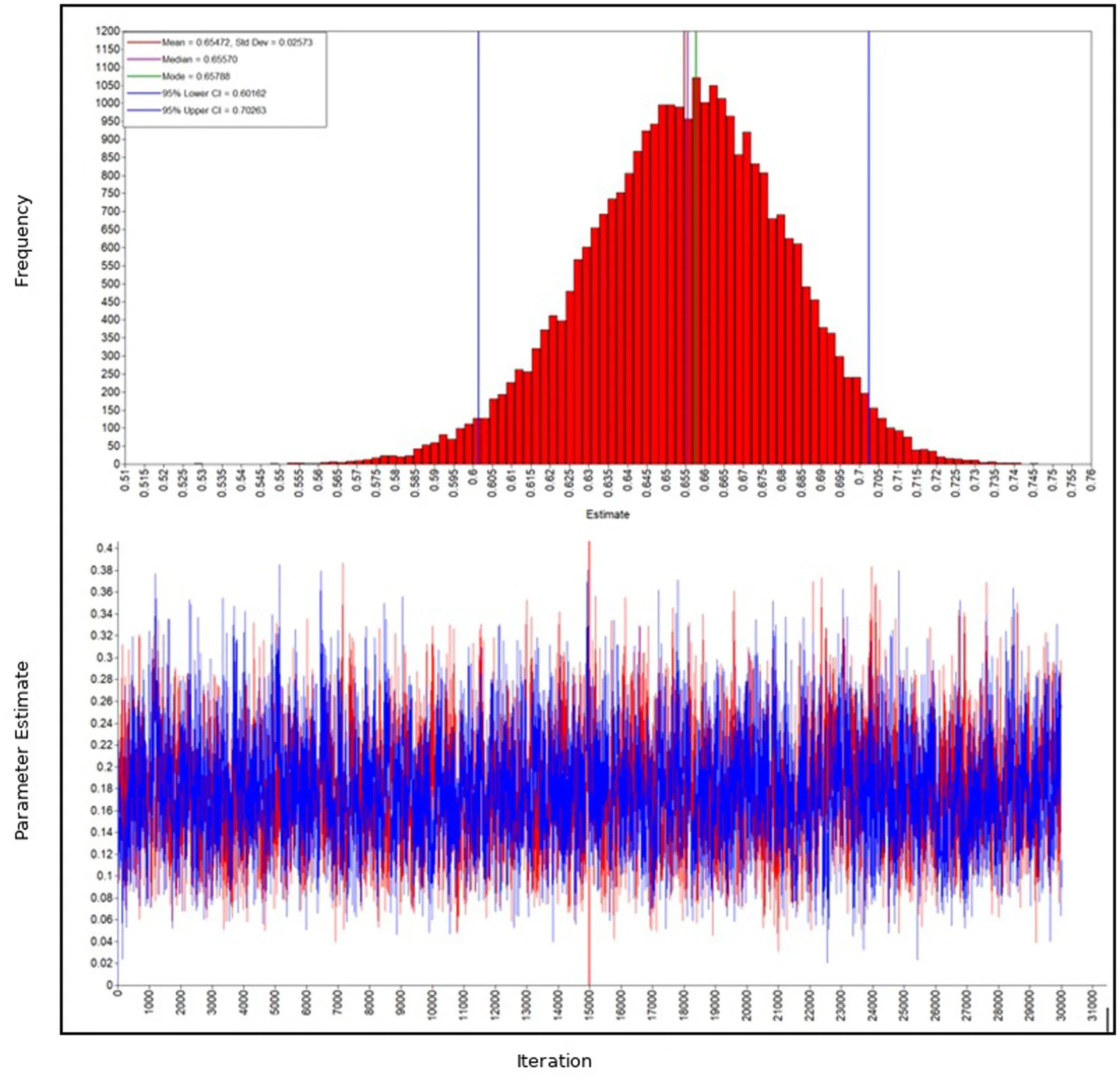
Trace plots and posterior distributions of the model parameters based on 70,000 MCMC iterations.

### 5.3 Direct and indirect relationships

Distributed leadership was positively associated with teachers’ subjective well-being [*β* = 0.233, 95% BCI (0.077, 0.381)], supporting H1. Distributed leadership was also positively associated with work engagement [*β* = 0.234, 95% BCI (0.135, 0.333)], supporting H2. In addition, teachers’ subjective well-being was positively associated with work engagement [*β* = 0.172, 95% BCI (0.112, 0.232)], supporting H3.

The indirect association between distributed leadership and work engagement through teachers’ subjective well-being was positive, and its 95% Bayesian credible interval excluded zero [*B* = 0.039, 95% BCI (0.012, 0.074)]. Therefore, H4 was supported, indicating a statistically credible indirect association between distributed leadership and work engagement through subjective well-being (Figure 3).

**Figure 3:**
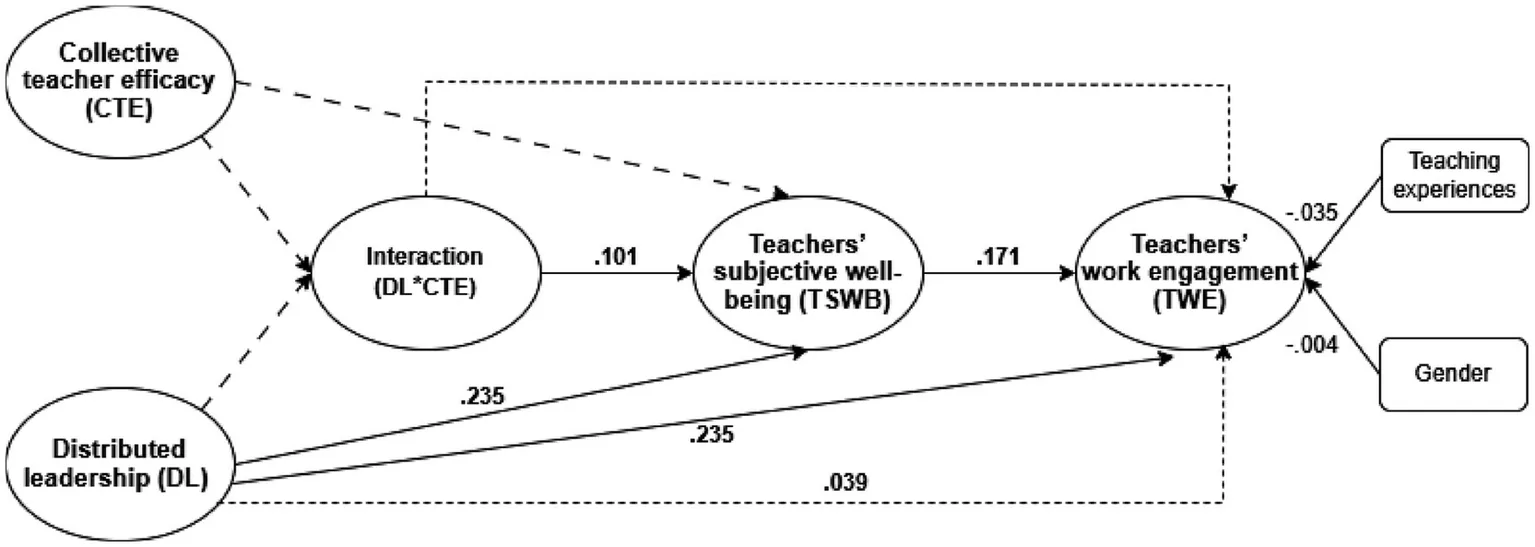
Bayesian structural model of the hypothesized moderated mediation relationships.

### 5.4 The moderating role of collective teacher efficacy

The interaction between distributed leadership and collective teacher efficacy was positive, and its 95% Bayesian credible interval excluded zero [*β* = 0.099, 95% BCI (0.003, 0.192)]. Thus, collective teacher efficacy moderated the association between distributed leadership and teachers’ subjective well-being, supporting H5.

Simple-slope analyses showed that the positive association between distributed leadership and subjective well-being became stronger as collective teacher efficacy increased. The association was positive at one standard deviation above the mean of collective teacher efficacy [+1 SD: *B* = 0.294, 95% BCI (0.101, 0.493)] and at its mean level [*B* = 0.206, 95% BCI (0.066, 0.350)]. At one standard deviation below the mean, however, the credible interval included zero [−1 SD: *B* = 0.119, 95% BCI (−0.014, 0.255)]. Accordingly, distributed leadership was positively associated with subjective well-being at average and high levels of collective teacher efficacy, whereas the evidence for this association was insufficient at its lower level (Figure 4).

**Figure 4:**
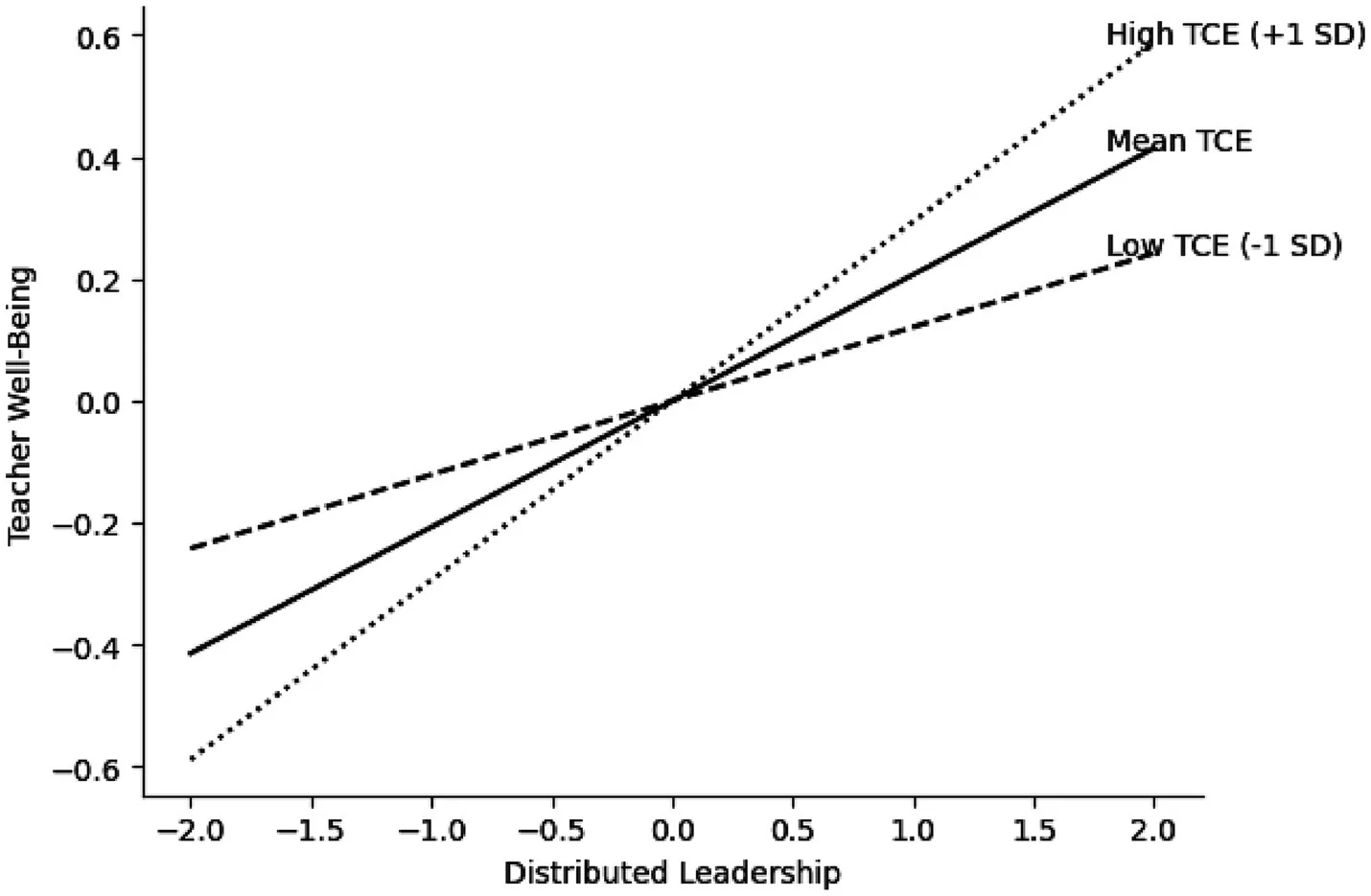
Association between distributed leadership and subjective well-being at different levels of collective teacher efficacy.

The conditional indirect association between distributed leadership and work engagement through teachers’ subjective well-being increased as collective teacher efficacy increased. The indirect association was supported at two standard deviations above the mean [+2 SD: *B* = 0.072, 95% BCI (0.021, 0.139)], one standard deviation above the mean [+1 SD: *B* = 0.056, 95% BCI (0.018, 0.105)], and the mean level of collective teacher efficacy [*B* = 0.039, 95% BCI (0.012, 0.074)]. In contrast, the credible intervals included zero at one standard deviation below the mean [−1 SD: *B* = 0.022, 95% BCI (−0.003, 0.053)] and two standard deviations below the mean [−2 SD: *B* = 0.006, 95% BCI (−0.029, 0.043)].

The index of moderated mediation was positive, and its 95% Bayesian credible interval excluded zero [*B* = 0.019, 95% BCI (0.001, 0.043)]. Therefore, the indirect association between distributed leadership and work engagement through subjective well-being varied across levels of collective teacher efficacy, supporting H6. Gender [*β* = −0.008, 95% BCI (−0.060, 0.044)] and teaching experience [*β* = −0.035, 95% BCI (−0.119, 0.021)] were not credibly associated with work engagement because their 95% Bayesian credible intervals included zero.

### 5.5 Prior sensitivity analysis

The robustness of the findings to alternative prior specifications was examined by re-estimating the structural model using weakly informative *N*(0,1) and broader *N*(0,10) priors. In both sensitivity models, normal priors with a mean of zero and variances of 1 and 10, respectively, were assigned to the structural coefficients. Both models terminated normally and demonstrated satisfactory convergence. In the *N*(0,1) model, the maximum PSR first fell below 1.05 at 56,200 iterations (PSR = 1.048) and reached 1.014 at the final 70,000th iteration. In the *N*(0,10) model, the maximum PSR first fell below 1.05 at 56,100 iterations (PSR = 1.049) and likewise reached 1.014 at the final iteration.

As shown in Table 2, the posterior estimates and 95% Bayesian credible intervals were virtually identical across the default, *N*(0,1), and *N*(0,10) prior specifications. The direction and interpretation of all direct, indirect, moderating, and conditional indirect associations remained unchanged. In particular, the interaction and index of moderated mediation continued to have credible intervals excluding zero, whereas the simple slope and conditional indirect associations at lower levels of collective teacher efficacy continued to have intervals including zero. These findings indicate that the substantive conclusions were not materially dependent on the prior specification.

**Table 2:** Prior sensitivity analysis for the Bayesian structural model.

Parameter	Default priors *B* [95% BCI]	*N*(0,1) *B* [95% BCI]	*N*(0,10) *B* [95% BCI]
DL → SWB	0.206 [0.066, 0.350]	0.205 [0.066, 0.349]	0.206 [0.066, 0.351]
CTE → SWB	0.158 [0.032, 0.286]	0.158 [0.032, 0.286]	0.158 [0.032, 0.286]
DL × CTE → SWB	0.102 [0.003, 0.209]	0.101 [0.004, 0.208]	0.102 [0.003, 0.209]
DL → WE	0.234 [0.135, 0.337]	0.235 [0.136, 0.338]	0.235 [0.135, 0.338]
CTE → WE	0.510 [0.411, 0.617]	0.509 [0.411, 0.616]	0.510 [0.411, 0.617]
SWB → WE	0.195 [0.124, 0.268]	0.195 [0.124, 0.268]	0.195 [0.124, 0.268]
Gender → WE	−0.014 [−0.102, 0.075]	−0.013 [−0.102, 0.075]	−0.014 [−0.102, 0.075]
Experience → WE	−0.019 [−0.030, 0.005]	−0.019 [−0.030, 0.005]	−0.019 [−0.030, 0.005]
Indirect association at mean CTE	0.039 [0.012, 0.074]	0.039 [0.012, 0.074]	0.039 [0.012, 0.074]
Index of moderated mediation	0.019 [0.001, 0.043]	0.019 [0.001, 0.043]	0.019 [0.001, 0.044]
Simple slope at +1 SD CTE	0.294 [0.101, 0.493]	0.292 [0.100, 0.491]	0.294 [0.101, 0.493]
Simple slope at mean CTE	0.206 [0.066, 0.350]	0.205 [0.066, 0.349]	0.206 [0.066, 0.351]
Simple slope at −1 SD CTE	0.119 [−0.014, 0.255]	0.119 [−0.014, 0.254]	0.119 [−0.014, 0.255]
Conditional indirect association at +2 SD CTE	0.072 [0.021, 0.139]	0.072 [0.021, 0.138]	0.072 [0.021, 0.139]
Conditional indirect association at +1 SD CTE	0.056 [0.018, 0.105]	0.055 [0.018, 0.104]	0.056 [0.018, 0.105]
Conditional indirect association at mean CTE	0.039 [0.012, 0.074]	0.039 [0.012, 0.074]	0.039 [0.012, 0.074]
Conditional indirect association at −1 SD CTE	0.022 [−0.003, 0.053]	0.022 [−0.003, 0.053]	0.022 [−0.003, 0.053]
Conditional indirect association at −2 SD CTE	0.006 [−0.029, 0.043]	0.006 [−0.029, 0.043]	0.006 [−0.029, 0.043]
Final maximum PSR	1.014	1.014	1.014

Entries are unstandardized posterior estimates with 95% Bayesian credible intervals, except for the final maximum PSR values. *N*(0,1) and *N*(0,10) denote normal priors with a mean of zero and variances of 1 and 10, respectively.

## 6 Discussion

This study examined how distributed leadership is associated with teachers’ work engagement through teacher subjective well-being and whether these associations vary according to perceived collective teacher efficacy. Drawing on distributed leadership theory, the JD-R model, and social cognitive theory, the study considered both the psychological pathway and the conditional association specified in the proposed model.

The findings indicate that distributed leadership is positively associated with teacher subjective well-being and work engagement. This result is consistent with the view that distributed leadership is embedded in social interactions and shared practices rather than confined to formal leadership roles (Spillane et al., 2004; Harris, 2013). Leadership practices characterized by participation, shared responsibility, and collaboration may function as organizational resources for teachers (Leithwood et al., 2009). In this sense, distributed leadership may be associated with professional environments in which teachers experience greater influence, recognition, and collegial support.

These findings are relevant to the KRG context, where school leadership has historically operated within centralized and hierarchical structures (Vernez et al., 2014). Although recent legislation has established formal structures for staff participation, teachers in Erbil have reported relatively limited experiences of distributed leadership (Demirtaş and Khudhur, 2026; Kurdistan Parliament, 2022). The present findings suggest that teachers’ experiences of participation and shared responsibility are associated with their well-being and engagement even within a predominantly centralized governance structure. However, the findings do not establish that distributed leadership has produced a broader structural change in KRG schools.

The results further show that subjective well-being is positively associated with work engagement and partially accounts for the association between distributed leadership and engagement. This finding is consistent with the JD-R model, which proposes that organizational resources are associated with positive psychological functioning and motivational outcomes (Bakker and Demerouti, 2017). Opportunities for participation, shared responsibility, and collegial support may be associated with teachers’ school connectedness and teaching efficacy, which may, in turn, relate to greater vigor, dedication, and absorption.

The partial nature of the indirect association is noteworthy because distributed leadership remained directly associated with work engagement after subjective well-being was included in the model. Subjective well-being therefore represents one possible psychological pathway rather than a complete explanation of the association between distributed leadership and engagement. Moreover, because the data are cross-sectional, the analysis cannot establish that subjective well-being temporally precedes work engagement. Reciprocal or alternative relationships between these constructs remain possible.

Perceived collective teacher efficacy moderated the positive association between distributed leadership and subjective well-being. This finding can be interpreted through social cognitive theory, which proposes that efficacy beliefs shape how people interpret and respond to environmental conditions (Bandura, 1997). When teachers perceive that teachers in their school can collectively address instructional and behavioral challenges, participation and shared responsibility may be accompanied by greater confidence in coordinated action and collective problem-solving. This may help explain why the association between distributed leadership and well-being was supported at the mean and higher levels of perceived collective teacher efficacy but not at its lower level.

This pattern is more consistent with an amplifying than a compensatory interpretation. Perceived collective capability appears to reinforce the organizational resources associated with distributed leadership rather than substitute for them. When perceived collective efficacy is low, the distribution of responsibility may not provide teachers with the same psychological benefits because participation is less likely to be accompanied by confidence in coordinated action.

This interpretation should nevertheless remain cautious. Collective teacher efficacy was measured through individual teachers’ perceptions and was not aggregated at the school level. The findings therefore concern differences among teachers in perceived collective efficacy and should not be interpreted as comparisons between objectively high- and low-efficacy schools. Leadership practices may also contribute to collective efficacy over time by creating opportunities for collaboration and shared success. Social cognitive theory allows for reciprocal relationships among beliefs, behavior, and environmental conditions (Bandura, 1997). Accordingly, treating perceived collective teacher efficacy as a moderator in the present model does not rule out the possibility that distributed leadership may also contribute to its development over time.

The moderated mediation results indicate that the indirect association between distributed leadership and work engagement through subjective well-being varied according to perceived collective teacher efficacy. The conditional indirect association was supported at the mean and higher levels of perceived collective efficacy but not at lower levels. Thus, the indirect association through subjective well-being appears stronger when participatory leadership practices coexist with stronger perceptions of collective capability.

The size of the index of moderated mediation was modest, and its credible interval was close to zero. The result should therefore be interpreted as evidence of a limited conditional pattern rather than a large difference across levels of perceived collective efficacy. The stability of the result under the alternative prior specifications strengthens confidence that it was not attributable solely to the default prior, but it does not overcome the limitations of the cross-sectional design.

Overall, the findings are consistent with previous research linking distributed leadership to teacher well-being, engagement, commitment, and related work outcomes (Bellibaş et al., 2024; Hulpia et al., 2009; Liu et al., 2021). The present study adds to this literature by examining subjective well-being as a possible psychological pathway between distributed leadership and work engagement and perceived collective teacher efficacy as a condition associated with the strength of that pathway. This responds to calls for closer examination of the mechanisms and conditions associated with distributed leadership (Tian et al., 2016).

### 6.1 Theoretical implications

The findings qualify the assumption that organizational resources are uniformly associated with teacher outcomes. Consistent with the JD-R model, distributed leadership was associated with work engagement both directly and indirectly through work-specific subjective well-being. This suggests that participatory decision-making, shared responsibility, and collaborative support may be related to engagement partly through teachers’ perceptions of their connection to the school and their effectiveness in teaching. However, the partial indirect association indicates that subjective well-being does not fully explain the relationship between distributed leadership and engagement.

Social cognitive theory helps explain why the association between distributed leadership and subjective well-being varied according to perceived collective teacher efficacy. Teachers reporting stronger confidence in the collective capability of teachers in their school also showed a stronger association between distributed leadership and well-being. The findings therefore point to an alignment between organizational resources and efficacy beliefs: participatory leadership may be more strongly associated with positive psychological functioning when teachers also believe that shared responsibilities can be enacted collectively.

The findings also clarify, but do not definitively resolve, the theoretical position of collective teacher efficacy. In the present model, perceived collective efficacy was treated as a condition shaping how teachers respond to distributed leadership. However, collective efficacy may also develop through shared decision-making, collaboration, and successful collective experiences. Future longitudinal research should therefore compare moderating, mediating, and reciprocal specifications rather than treating collective efficacy as having a single fixed role.

### 6.2 Practical implications

For teachers, the findings point to the potential value of meaningful participation in school decisions, shared responsibility, and collaborative problem-solving. Such participation is more likely to be meaningful when teachers have genuine professional influence rather than simply additional administrative duties. Working toward shared goals and reviewing collective accomplishments may also help teachers develop confidence in their collective capability.

For school principals, distributed leadership may require more than forming committees or delegating tasks. Principals could provide teachers with a meaningful voice in decisions, clarify responsibilities, encourage mutual support, and provide the authority and resources needed for participation. Because the association between distributed leadership and well-being varied modestly according to perceived collective teacher efficacy, opportunities for joint problem-solving, constructive feedback, and achievable collaborative work may complement participatory leadership practices. However, the small conditional effect does not support treating collective efficacy as a prerequisite for successful distributed leadership.

For policymakers in the KRG, the findings suggest that formal participatory structures alone may not ensure that teachers experience meaningful influence. Implementation efforts could include professional development for principals in shared decision-making and collaborative leadership, alongside opportunities for schools to develop collective capacity. These implications should be interpreted cautiously because the conditional effect was modest, the study was limited to teachers in Erbil, and no particular policy or leadership intervention was evaluated.

## 7 Limitations and future research

This study has several limitations. First, its cross-sectional design does not establish causal relationships or the temporal ordering assumed in the mediation model. Alternative and reciprocal relationships among distributed leadership, perceived collective teacher efficacy, subjective well-being, and work engagement remain possible. Future longitudinal studies should examine how these relationships develop over time. Accordingly, the reported mediation represents a statistical decomposition of covariance among simultaneously measured variables rather than evidence of a causal mechanism or temporal sequence. The proposed direction from distributed leadership to work engagement through subjective well-being was theoretically specified but was not empirically compared with alternative directional models.

Second, all focal variables were assessed through teacher self-reports at a single time point. Harman’s test and the CFA-based single-factor comparison indicated that a single factor did not adequately account for the covariance among the measures. However, these statistical diagnostics cannot completely rule out common method bias. Future studies could obtain information from multiple sources and separate the measurement of predictors, mediators, and outcomes across time.

Third, convenience sampling and the focus on public-school teachers in Erbil limit the generalizability of the findings. Because the total number of teachers who received the survey link was not recorded, the response rate could not be calculated. Distribution through school communication networks may also have introduced self-selection or socially desirable responding, particularly because the questionnaire included perceptions of school leadership. Probability-based sampling and the inclusion of other cities would provide a stronger basis for conclusions about the wider KRG education system.

Fourth, although participants were drawn from 60 schools, individual responses could not be linked to particular schools. Consequently, school-level clustering, intraclass correlations, and between-school variation could not be examined. Perceived collective teacher efficacy was analyzed at the individual teacher level and should not be interpreted as an aggregated school characteristic. Future studies should retain anonymized school identifiers and use multilevel models to distinguish individual perceptions from shared school-level efficacy.

Fifth, the operationalizations of the constructs have relevant scope limitations. The distributed leadership measure primarily captures participatory decision-making, shared responsibility, and collaborative culture rather than every relational and practice-based dimension of distributed leadership. Teacher subjective well-being was operationalized specifically through school connectedness and teaching efficacy. The conceptual proximity between individual teaching efficacy and perceived collective teacher efficacy should also be considered, although the HTMT findings supported their empirical distinctiveness in the present sample.

Finally, the instruments were administered in English, which may not have been the first language of all participants. Although the CFA results supported their factorial structures in this sample, they do not establish linguistic equivalence or rule out differences in item interpretation arising from English proficiency. The restricted range of teaching experience, from 2 to 16 years, may also limit the applicability of the findings to teachers with longer professional careers. Future studies could examine linguistic equivalence more directly and include teachers with a broader range of teaching experience.

## 8 Conclusion

This study found that distributed leadership was associated with teachers’ work engagement both directly and indirectly through teacher subjective well-being. The indirect association varied according to perceived collective teacher efficacy and was supported at the mean and higher levels of the moderator but not at lower levels. These findings indicate an indirect statistical association between participatory leadership practices and work engagement through teachers’ positive work-specific functioning, with the magnitude of this association varying according to perceived collective capability.

In the Kurdistan Region of Iraq, where formal opportunities for participation coexist with centralized and hierarchical governance, the findings draw attention to the difference between establishing participatory structures and ensuring that teachers experience meaningful influence and shared responsibility. Nevertheless, the results are based on cross-sectional, individual-level perceptions from teachers in Erbil and should not be interpreted as causal evidence or generalized to all schools in the KRG.

## Data Availability

The raw data supporting the conclusions of this article will be made available by the authors, without undue reservation.
